# Unveiling the Growth Impact of Probiotics in Neonates: To BEgin or Not to BEgin?

**DOI:** 10.3390/nu17111867

**Published:** 2025-05-29

**Authors:** Mustafa Törehan Aslan, Yasemin Ersözlü, Metehan Özen

**Affiliations:** 1 Department of Neonatology, Koç University Hospital, 34010 Istanbul, Türkiye; torehanaslan@yahoo.com; 2 Department of Pediatric Infectious Disease, School of Medicine, Acibadem University, 34752 Istanbul, Türkiye; yaseminersozlu@gmail.com

**Keywords:** probiotic, dietary patterns, diet quality, neonatal care, newborn, weight gain

## Abstract

**Background/Objectives:** This study aimed to evaluate the supportive role of probiotic supplementation in neonatal weight gain through a meta-analysis of published studies. Given the conflicting results in the literature, the objective was to determine the overall effect size and assess the influence of regional and intervention-specific factors. **Methods:** A total of 20 studies published between 2011 and 2022 were included, comprising a combined sample size of 3929 neonates. A random-effects model was used to calculate the pooled standardized mean difference (SMD) in neonatal weight gain attributable to probiotic supplementation. Heterogeneity among studies was assessed using the *I*^2^ statistic. Subgroup analyses were conducted based on geographic region, probiotic strain, dosage, and treatment duration. **Results:** The pooled analysis demonstrated a modest but non-significant positive effect on neonatal weight gain (SMD: 0.27; 95% CI: −0.06 to 0.61), with substantial heterogeneity across studies (*I*^2^ = 91%). Subgroup analyses indicated that regional variations, particularly in studies conducted in China, were associated with a more favorable effect. However, not all studies reported a benefit; some found no difference or even negative effects, particularly in discharge weight outcomes. **Conclusions:** Probiotic supplementation shows potential for improving neonatal weight gain, but findings remain inconsistent and heterogeneous. Strain selection, dosage, and treatment duration appear to be critical variables influencing outcomes. Future large-scale, multicenter randomized controlled trials are necessary to develop standardized, evidence-based guidelines for probiotic use in neonatal care.

## 1. Introduction

In the past few years, probiotics have gained a lot of attention for their use in neonates; this is because they may help to promote their gut health, prevent infection, and boost their immune system. Neonatal care has turned into a series of interventions, which aim to improve the health of newborns, especially premature or vulnerable newborns [1]. Probiotics—live microorganisms that confer health benefits—are increasingly considered as supportive adjuncts to promote neonatal health, rather than direct therapeutic agents in the classical sense. Despite that, however, there remains vigorous debate about whether to introduce probiotics to neonates, particularly to those in intensive care [2]. Probiotic supplementation is being explored as a supportive measure, particularly in preterm neonates due to their susceptibility. However, the question of whether or not this population might have an insufficient long-term efficacy and safety of the probiotic itself will require additional study before broad recommendation.

The problem is, there is no agreement between health professionals in using probiotics in neonates, especially as it pertains to preterm and low weight neonate who are vulnerable to complications like necrotizing enterocolitis (NEC), a severe condition of the small intestine that frequently leads to extensive damage [3]. However, some studies suggest that probiotics may lower the incidence of NEC and other GI disorders, but they raise concern regarding the safety of probiotics use in immunocompromised neonates due to the risk of infections [4]. In addition, probiotic strains, dosage, and duration of treatment have been standardized poorly. Since probiotics are of special interest in this very vulnerable population, we must investigate the safety and efficacy of probiotics in this group to provide evidence-based guidance for probiotics use [5].

Preterm infants are particularly vulnerable to complications such as feeding intolerance, gut immaturity, and microbial dysbiosis—conditions strongly linked to underdeveloped intestinal microbiota and impaired immune regulation [6]. Probiotics have emerged as a promising adjunct strategy to support microbial balance, enhance mucosal integrity, and reduce inflammation and infection risk in this population [1]. However, before probiotics can be routinely incorporated into neonatal care, their efficacy and safety must be supported by high-quality, evidence-based research. It is essential to comprehensively assess both the potential benefits and risks associated with probiotic use in neonates, particularly those who are medically fragile. Robust scientific data are needed to inform clinical decision-making, shape guideline development, and support discussions with caregivers regarding the use of probiotics in neonatal care.

This study presents a comprehensive meta-analysis aimed at systematically evaluating the impact of probiotic supplementation on neonatal health, with a specific focus on weight gain. Meta-analysis provides a rigorous statistical framework for synthesizing data across multiple independent studies, thereby enhancing statistical power and improving the precision of effect estimates. By integrating evidence from both randomized controlled trials and observational studies published between 2011 and 2022, this analysis seeks to identify consistent trends, assess sources of variability, and offer a broader understanding of probiotic efficacy and safety in neonates [7]. Given the diversity in study designs, probiotic strains, dosages, and outcome definitions, meta-analytic techniques are well suited to address inconsistencies in the existing literature and to generate more reliable, generalizable conclusions. Furthermore, this approach allows for stratified analyses based on key factors such as probiotic type, administration timing, and geographic context, providing clinically relevant insights that can inform neonatal care practices and guide future research and policy development.

## 2. Methods

### 2.1. Study Design

The purpose of this meta-analysis is to evaluate the effect of probiotics on neonatal weight gain from randomized controlled trials (RCTs) and observational studies, published between 2011 and 2022. The outcome variable of the included studies refers to the effects of probiotic interventions on neonates’ weight gain. The studies were of different regions and countries or of different probiotic strains and dosages. This study follows a systematic and quantitative approach to merging individual studies results into a single summary estimate, thus, providing a comprehensive synthesis of the pooled evidence of the efficacy of probiotics to increase neonatal weight gain.

### 2.2. Search Strategy

A comprehensive and systematic literature search was done to identify the relevant studies for this meta-analysis. Electronic databases searched were PubMed, Cochrane Library, Scopus, and Google Scholar. Studies published between 2011 and 2022 were included in the search to also include recent findings. The following keywords and phrases were used in the search: ‘probiotics’, ‘neonates’, ‘weight gain’, ‘randomized controlled trials’, ‘preterm infants’, ‘gastrointestinal health’, ‘*Bifidobacterium*’, ‘*Lactobacillus*’, and specific probiotic strains employed in the included studies, like ‘*Lactobacillus reuteri*’, ‘*Lactobacillus acidophilus*’, and ‘*Bifidobacterium bifidum*’.

### 2.3. Inclusion and Exclusion Criteria

In this meta-analysis, only studies satisfying certain criteria were included to perform an entire evaluation of the effect of probiotics on neonatal weight gain (Table 1). The study population was composed of both term and preterm neonates undergoing probiotics as part of their care and focused specifically on weight gain; both neonates and mothers who received probiotics. Studies of neonates with gastrointestinal disorders, such as necrotizing enterocolitis or feeding intolerance, were also included, as these diseases are related to the assessment of probiotic efficacy.

The interventions conducted in these studies consisted of either oral or enterally administrating of probiotics to neonates. Some probiotic strains included in this were of single-strain and multi-strain, *Lactobacillus reuteri*, *Bifidobacterium* species, *Saccharomyces boulardii*, etc. Neonatal weight gain was measured as the primary outcome and was assessed at various points during the study, for instance, on consecutive days or weekly.

RCTs and observational studies are the study designs considered, the latter being preferred for being less rigorous than RCTs. To widen the knowledge of probiotics effects, additional non-randomized studies were included. Articles and studies presenting neonates over six months of age or indicating weight gain as an outcome that did not report weight gain as an outcome were excluded, and those articles and studies published in peer-reviewed journals alone were included. Additionally, studies lacking in probiotic strain, dose and outcome information were excluded from analysis.

### 2.4. Article Screening

In two phases, article screening was carried out to ensure the inclusion of high-quality studies. The titles and abstracts of all retrieved articles were first independently reviewed by two researchers, who then independently conducted an initial review of all these identified articles. At this stage, all studies which did not address the primary focus on neonatal care, probiotics or weight gain outcomes were excluded. Then any duplicates or irrelevant articles were also removed.

The second phase was the retrieval and assessment of full-text articles of potentially eligible studies against the inclusion and exclusion criteria. The false results were disputed by both researchers and resolved through discussion or with a third reviewer’s input. Articles that did not report on weight gain as an outcome, addressed a population older than six months, or did not have sufficient information on probiotics, dosage and outcome measures were excluded. As part of this rigorous screening, only studies of high relevance and robust data were included in the meta-analysis.

### 2.5. Data Extraction

Two independent reviewers extracted data in a consistent and bias-minimizing form. The extracted key information contained study characteristics (authors, year, country, study design), participant details (sample size, gestational age, sex distribution) and intervention specifics (probiotic strain, dosage, administration method, duration). The relevant time points for outcome measures (mean, standard deviation) were recorded for neonatal weight gain. The difference between reviewers was arbitrated during discussion with or by a third reviewer until no differences remained. In order to represent the studies accurately for the meta-analysis, a thorough data extraction process was conducted in order to extract the data in such a way.

### 2.6. Statistical Analysis

For this meta-analysis, the statistical analysis was performed with R-Studio, a very powerful statistical computing environment that is extremely popular for the conduct of meta-analyses. The aim was to evaluate neonatal weight gain, which had been investigated in many studies. In order to calculate the pooled effect size, we used the mean difference (MD) to compare weight gain between the probiotic and control groups. Pooled effect was additionally estimated by calculating the 95% CIs to see how precise the pooled effect was.

Because the included studies are expected to be heterogeneous, a random-effects model was used. This model incorporates within and between study variability of effect size to obtain more conservative effect size estimates. The *I*^2^ statistic calculated the proportion of total variation across studies due to heterogeneity, rather than due to chance. Subgroup analyses were done to explore possible sources of significant heterogeneity based on the finding of an *I*^2^ value greater than 50%, indicating significant heterogeneity.

Finally, sensitivity analyses were performed to ascertain the robustness of the findings. This process involved the systematic removal of each study and evaluating the impact of individual studies on the overall pooled effects. Funnel plots were used to assess potential publication bias as well. In case publication bias was detected, subsequent adjustments were performed with the trim and fill method.

## 3. Results

### 3.1. Study Selection

**Figure 1:**
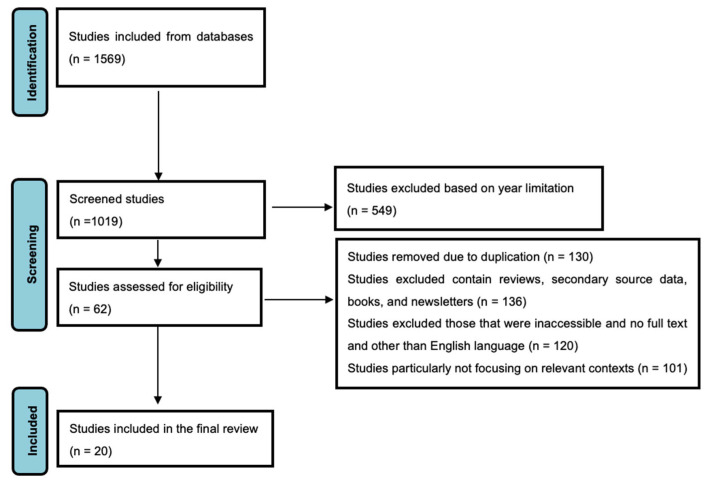
PRISMA Flow Diagram Depicting the Selection Process of Studies Included in the Meta-Analysis.

This meta-analysis was carried out systematically by selecting the eligible studies for this meta-analysis (Figure 1). To begin, 1569 studies were identified through searches of databases. From 322 titles and abstracts screening, 1019 studies were reviewed for relevance. Based on pre-defined criteria, 62 studies were assessed for their eligibility of being based on probiotics and neonatal weight gain. At this stage, several studies were excluded (549 studies), duplicate (130 studies), review (136 studies), or other (secondary data, non-peer-reviewed sources, or not accessible due to language issues) (120 studies). A robust dataset for analysis was available in the case of the final set of 20 studies (total sample size, *n* = 3929) meeting all inclusion criteria.

### 3.2. Study Characteristics

This meta-analysis included studies from countries located on different continents, like Europe, Asia, Africa, Australia, and North America. The sample sizes were 35 to 1099 (total sample size, *n* = 3929) neonates and the primary outcome measure was weight gain. Interventions with probiotics were often single-strain, characterized by *Lactobacillus* or *Bifidobacterium* species, or multi-strain, including *Lactobacillus rhamnosus* GG and *Saccharomyces boulardii*. In this period, several studies were published from 2011 up to 2022 reporting on weight gain and a few others reporting on weight at discharge (Table 2). Different interventions were used across a range of neonates, i.e., term and preterm infants.

### 3.3. Forest Plot and Sub-Group Analysis

**Figure 2:**
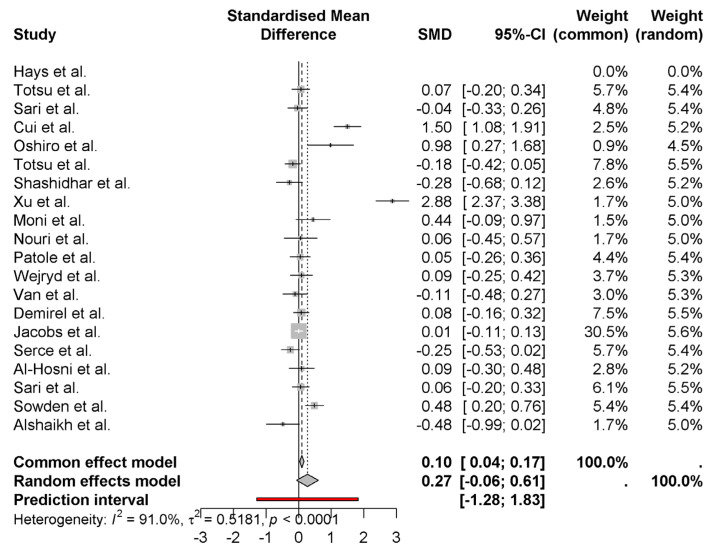
Weight gain outcomes among neonates on probiotics [8,9,10,11,12,13,14,15,16,17,18,19,20,21,22,23,24,25,26,27].

The forest plot provides the standardized mean differences (SMD) of weight gain outcomes among neonates on probiotics in various studies. A small positive effect of probiotics on weight gain by 0.27 (95% CI: −0.06 to 0.61) using the random effects model in the pooled overall estimate. It is high heterogeneity (*I*^2^ = 91.0%), which means that there is lots of variation across studies (Figure 2). As is the case for Xu et al. [15] (2.88) and Sari et al. [10] (1.50), large amounts of effects are seen in some studies, while others such as Alshaikh et al. [27] (−0.48) feature negative effects. It is evident that the uncertainty in the individual study results is considerable, as judged from the wide prediction interval (−1.28 to 1.83).

**Figure 3:**
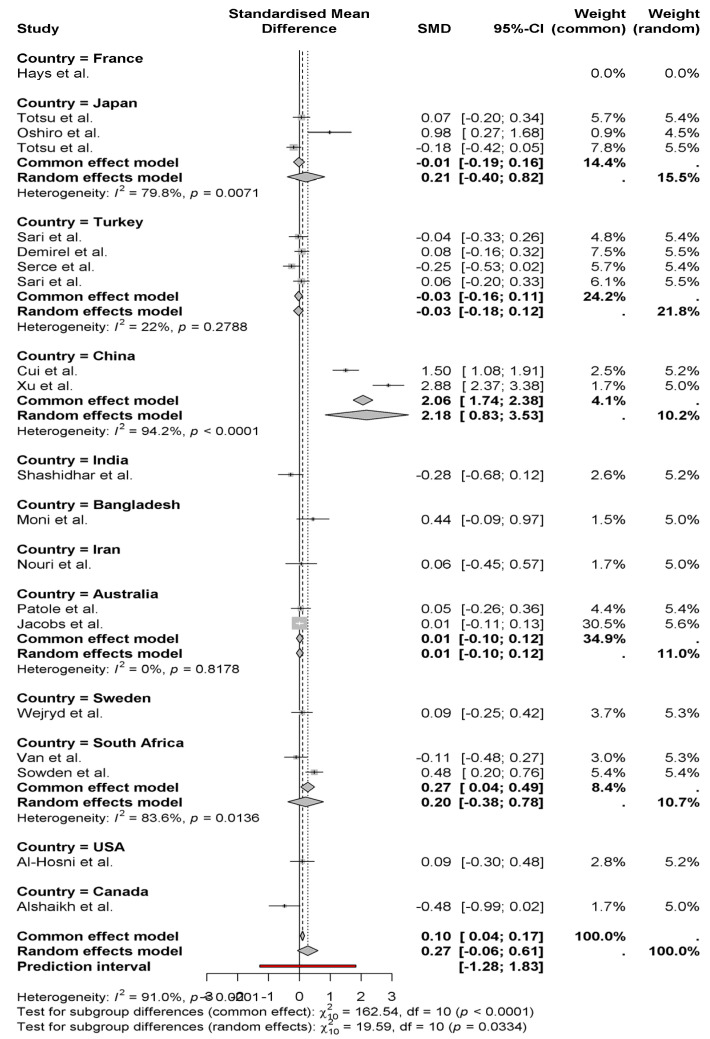
Weight gain between neonates who received probiotics, which are arranged by national origin [8,9,10,11,12,13,14,15,16,17,18,19,20,21,22,23,24,25,26,27].

The forest plot contains Standardized Mean Differences (SMD) measurements of weight gain between neonates who received probiotics, which are arranged by national origin. According to the random effects model, the combined analysis generates a modest positive outcome of 0.27 for weight gain in newborns (95% CI: −0.06 to 0.61) (Figure 3). Studies show large differences in outcomes between studies (*I*^2^ = 91%), which mainly stem from variations in their specific regions. Research conducted in China yielded the most substantial positive effects of probiotics (2.06 and 2.88 SMDs) according to Cui et al. [11] and Xu et al. [15]. As reported by Alshaikh et al. [27] in Canadian research, the results show a negative weight impact (−0.48). Research findings differ between countries, possibly due to regional elements which include probiotic strain types as well as dosage and experimental methodology. The statistical subgroup analysis indicates substantial differences between regions, which demonstrates the necessity to investigate these elements more deeply.

**Figure 4:**
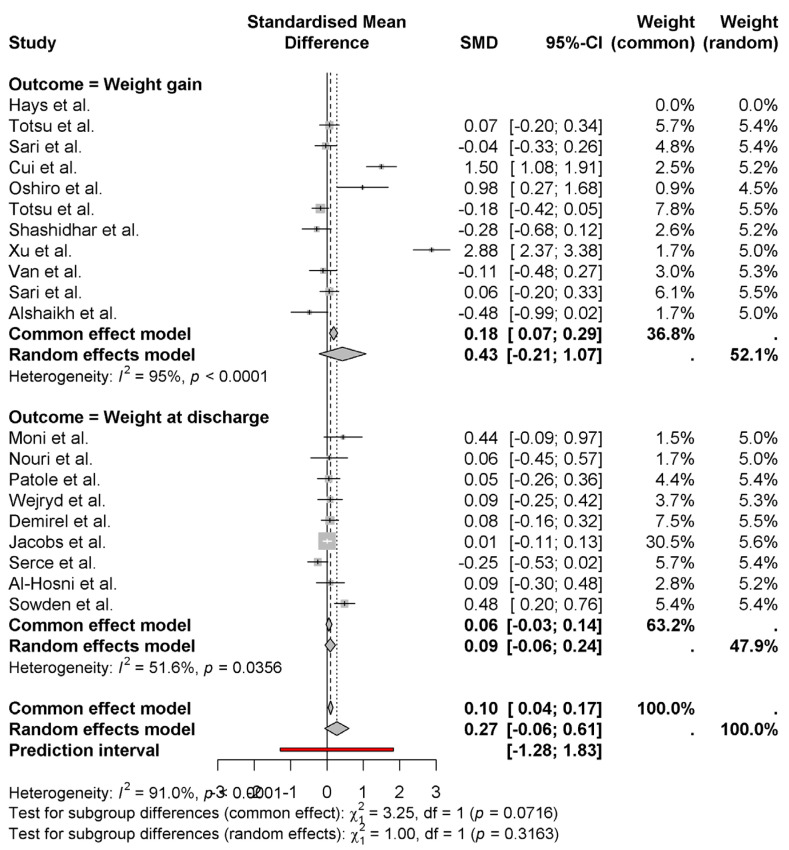
Effects of probiotics on weight gain and discharge weight of newborns [8,9,10,11,12,13,14,15,16,17,18,19,20,21,22,23,24,25,26,27].

The pooled effect of probiotics on neonatal weight gain and weight at discharge using common (fixed) and random effects models is represented as a forest plot. The random effects model indicated that probiotics result in a moderate positive effect for weight gain (SMD = 0.43 0.21 to 1.07), with moderate heterogeneity (*I*^2^ = 95%) (Figure 4). These studies, such as those by Cui et al. [11] (1.50) and Xu et al. [15] (2.88), have a larger positive effect; others, such as Alshaikh et al. [27] (−0.48), have a negative effect. The pooled effect size for weight at discharge is smaller (SMD = 0.09, 95% CI: −0.06 to 0.24) and with some moderate heterogeneity (*I*^2^ = 51.6%). The results of subgroup analysis by country show no significant differences in the outcomes between studies (*p* = 0.0716 for common (fixed) effect, *p* = 0.3163 for random effect). Given these findings, probiotics may modestly affect weight gain but not weight at discharge, and study variation is large.

**Figure 5:**
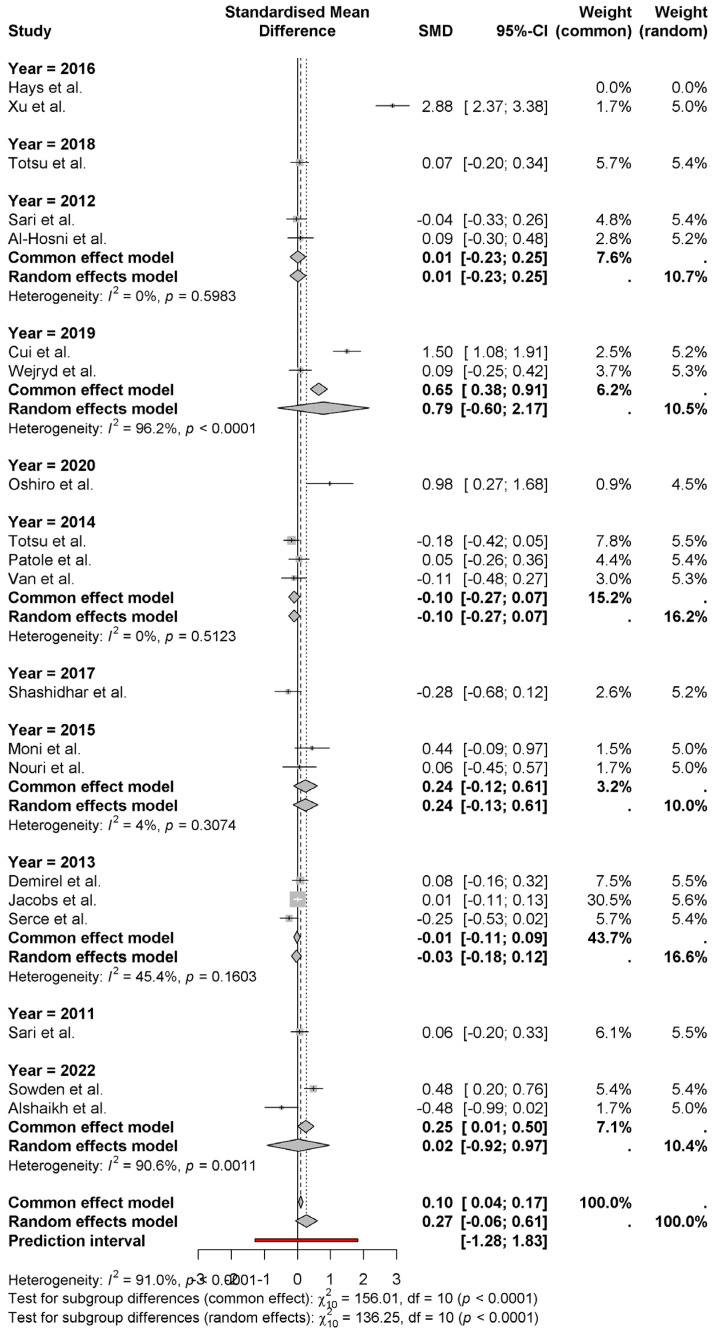
The standardized mean differences (SMD) for neonatal weight gain, grouped by publication year [8,9,10,11,12,13,14,15,16,17,18,19,20,21,22,23,24,25,26,27].

The forest plot shows the standardized mean differences (SMD) for neonatal weight gain grouped by publication year. The analysis from the random-effects model provides a pooled effect size of 0.27 (95% CI: −0.06 to 0.61) with significant heterogeneity (*I*^2^ = 91.0%) (Figure 5). In 2016, studies of Hays et al. [8] and Xu et al. [15] reported the largest positive effect (SMD = 2.88 and 1.50, respectively). However, 2022 (Alshaikh et al.) [27] studies show a negative (−0.48) effect. It shows substantial variation in effect sizes across years, and studies from 2019 (Cui et al.) [11] and 2017 (Shashidhar et al.) [14] have a moderate positive effect by showing some effect. Analysis of subgroup reveals that studies conducted in different years show significant differences in outcome (*p* < 0.0001); therefore, it suggests that other factors such as study design, intervention details or population characteristics may account for observed effects. These factors show the need for further research to clarify these.

**Figure 6:**
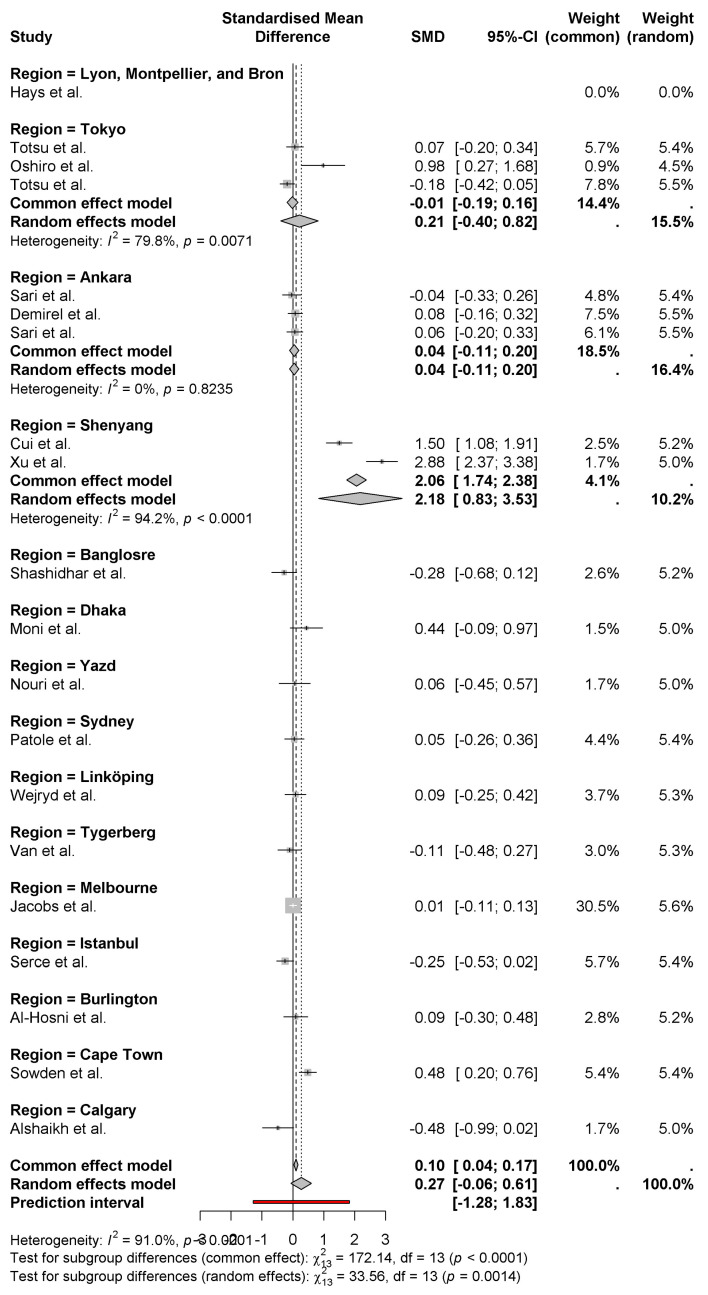
Differences in weight gain of newborns by region [8,9,10,11,12,13,14,15,16,17,18,19,20,21,22,23,24,25,26,27].

The forest plot shows the standardized mean differences (SMD) of neonatal weight gain by region. A pooled effect size of 0.27 [95% (CI) −0.06 to 0.61] is revealed using the random-effects model that probiotics have a modest positive effect on neonatal weight gain. Unequal heterogeneity (*I*^2^ = 91%) denotes high variation in the results in the regions (Figure 6).

ShenYang regions (Cui et al. and Xu et al.) [11,15] have the largest positive effects with SMDs of 2.06 and 2.88, respectively. In contrast, regions like Istanbul (Serce et al.) [23] and Calgary (Alshaikh et al.) [27] show negative effects of −0.25 and −0.48, respectively. Further subgrouping analysis demonstrates regional variation in the effectiveness of probiotics, and regions significantly differ in common (fixed) and random effects models (*p* < 0.001). This demonstrates the effect of regional factors (i.e., probiotic strains) and demographic variations in populations.

**Figure 7:**
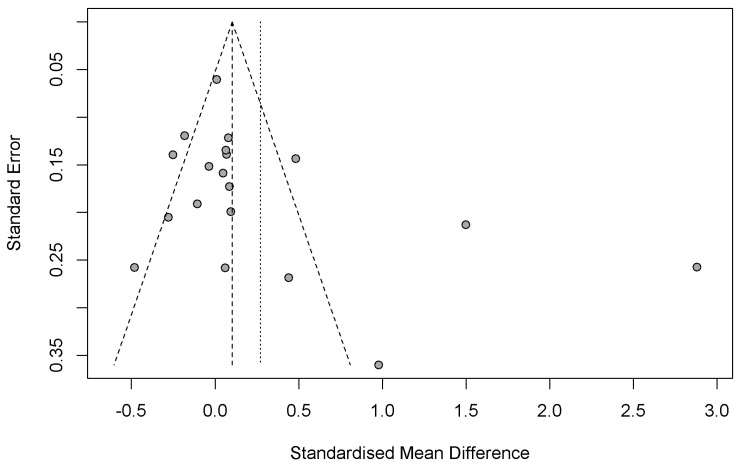
Funnel plot for evaluating potential publication bias and heterogeneity.

Figure 7 presents the funnel plot used to evaluate the presence of potential publication bias among the studies included in the meta-analysis. The vertical axis represents the standard error of individual studies, while the horizontal axis depicts the standardized mean difference (SMD). In an unbiased distribution, studies are symmetrically scattered around the mean effect size, forming an inverted funnel shape.

In this plot, the majority of studies are clustered near the top, indicating smaller standard errors and larger sample sizes. These studies are generally centered around the null effect, suggesting consistent findings with minimal deviation. However, a noticeable asymmetry exists, with an apparent absence of studies on the left side of the mean (negative SMD values). This imbalance suggests the potential presence of publication bias, where studies reporting non-significant or negative results are less likely to be published or included.

Additionally, a few studies are observed far from the center with large positive effect sizes, possibly indicating heterogeneity in the data. The dashed lines represent the expected 95% confidence limits in the absence of bias, while the vertical dotted line shows the estimated mean effect size. Collectively, these observations point to potential bias and variability that should be considered when interpreting the meta-analytic findings.

## 4. Discussion

A meta-analysis was conducted on 20 reported studies (total sample size, *n* = 3929) involving the use of probiotics on neonatal weight gain from around the globe. Within the random effects model, the overall pooled effect size for weight gain was 0.27 (95% CI: −0.06 to 0.61), which was a modest positive effect of probiotics on neonatal weight gain. Though heterogeneity was quite high (*I*^2^ = 91%), this indicated heterogeneity between studies. Regarding subgroup analyses, studies conducted in China [11,15] and Canada [27] indicate different positive and some even negative effects. While subgroup analysis revealed stronger positive effects in certain geographic regions such as China, we acknowledge that this may reflect region-specific microbiome profiles, clinical protocols, and strain usage rather than a universal effect. Therefore, the generalizability of these findings across regions should be interpreted with caution. Further, globally diverse multicenter trials are needed to validate region-specific outcomes. The forest plot also brings to light that those studies from more recent years yielded positive results, and that studies of earlier years present a wider diversity of outcomes. Publication bias could not be denied because of asymmetry in the funnel plot analysis, which indicated the possibility of the underreporting of studies with non-significant or negative results. The findings also indicate that probiotics influence neonatal weight gain in a positive way, but the impact may differ in the regions and in the studies. The considerable heterogeneity (*I*^2^ = 91%) across studies may be attributed not only to clinical and methodological diversity, but also to biological variability in host-microbiome interactions. Strain-specific differences in colonization efficiency, immunomodulatory properties, and metabolite production may alter the impact of probiotics on nutrient absorption and metabolic regulation. Furthermore, population-level factors such as delivery mode, antibiotic exposure, and regional microbiome composition likely contribute to differential probiotic responses [2,28]. These observations underscore the importance of exploring mechanistic pathways and individualized biological responses in future probiotic research, with an emphasis on their supportive and modulatory roles rather than direct therapeutic effects. The factors to consider here may include probiotic strain, dosage, study design and population characteristics. There is a need for further research to identify the best probiotic strains and doses for neonates.

This meta-analysis has two corroboratory studies in recent years that show how probiotics have beneficial effects on neonatal weight gain. Wang et al. [29] performed a systematic review and network meta-analysis of over 25,000 preterm infants which showed that multiple strain probiotics were related to reduce all-cause mortality, NEC, feeding intolerance and hospitalization. A similar study conducted by Panchal et al. [30] found that preterm infants supplemented with probiotics gained better short-term weight with a standardized mean difference (SMD) of 0.24. Additionally, Han et al. [28] conducted a systematic review including 35 studies finding a significant decrease of the rate of NEC and related mortality in preterm infants by probiotic supplementation. These results fit the current meta-analysis, a finding modest positive effect of probiotics on neonatal weight gain. Together, these studies emphasize the capability of probiotics to improve the neonatal growth outcomes, especially in preterm infants. Although our population included both term and preterm neonates, the majority of studies predominantly enrolled preterm infants, who inherently present greater susceptibility to growth restriction. Thus, our conclusions may be more applicable to this subgroup, although we refrained from conducting subgroup-specific meta-analyses to preserve statistical power and minimize selection bias. The strength of the evidence supporting probiotic supplementation in neonatal care comes from the consistency of these findings across different populations and methods of research.

This meta-analysis has some findings that are in disagreement with those reported by several studies that analyzed probiotics in neonatal weight gain. In fact, a systematic review and meta-analysis by Rasaei et al. [31] showed that probiotics are not effective at reducing the weight gain of very low birth weight (VLBW) infants in the short term. Like in Aslamzai et al. [32], a study has shown no difference in body weight between probiotic and placebo groups at 18 months of age despite short-term benefits. These results underscore the importance of additional work to disentangle the factors influencing such effects and show that probiotics cannot be easily assessed as an effect on neonatal weight gain. However, due to heterogeneity in the studies and lack of research in this area, there is a need to conduct further research as well in this field to provide robust policy implications.

## 5. Policy Implications

These findings have important policy-related implications for neonatal care as they relate to the use of probiotics in neonates. The two most important implications are, first, that standardized guidelines for probiotic supplementation should be developed for use in neonatal care and, second, that there should be large, randomized controlled trials aimed at providing evidence to support such guidelines. With a very modest positive effect on weight gain in the studies, policymakers should aim to create uniform protocols laying out which probiotic strains, dosages, and treatment durations make sense for which neonatal population. Given that neonates require consistent and effective care at all health care settings, there should be guidelines based on the updated evidence. In addition, the large heterogeneity of study outcomes suggests the requirement of additional large, multi-center studies to assess probiotics efficacy and safety for neonates. There is an opportunity for makers of policy to fund and support such research to meet gaps in the knowledge of how probiotics impact the growth and development of neonates over their lifespan. In addition, because probiotics are not universally regulated for use in neonates, there is a pressing need for enhanced regulation. The requirement for probiotics used in neonates to meet extremely safe standards should be pushed strongly by policymakers. This will encompass developing processes to certify the probiotic products that are used for the neonates and the need to ensure their quality, purity, and efficacy.

## 6. Conclusions

This meta-analysis identified a modest, though inconsistent, positive effect of probiotic supplementation on neonatal weight gain. However, significant heterogeneity limits the generalizability of these findings. Until robust, standardized, and long-term trials are conducted, routine probiotic administration in neonatal care should be approached cautiously. Future research should focus on strain-specific efficacy, long-term safety, and personalized microbiome-targeted strategies to optimize neonatal outcomes.

### Limitations and Future Directions

This meta-analysis provides valuable insights into the effects of probiotic supplementation on neonatal weight gain; however, several limitations must be acknowledged when interpreting its findings. A substantial degree of heterogeneity was observed among the included studies (*I*^2^ = 91%), which limits the overall generalizability of the results. This variability likely stems from differences in probiotic strains, dosages, treatment durations, study populations, and methodological designs. Such heterogeneity complicates efforts to identify the most effective probiotic formulations and regimens for neonatal use. In addition, several studies included in the analysis had relatively small sample sizes, which may have introduced sampling bias and reduced the overall statistical power of the meta-analysis. While our study population encompassed both term and preterm neonates, the findings are likely more representative of preterm infants, given their predominance and heightened vulnerability to growth impairment. Subgroup analyses were not performed to avoid compromising statistical power and increasing the risk of bias. Although some randomized controlled trials (RCTs) were included, they represented a minority compared to observational studies. Given that RCTs remain the gold standard for evaluating causality, the predominance of observational data reduces the strength of causal inference in this review. Another key limitation is the lack of long-term outcome assessments in most studies. Important endpoints—such as neurodevelopmental milestones, cognitive performance, and immune system modulation—were seldom reported, despite their relevance in evaluating the broader and sustained impacts of early-life probiotic use. Future research should prioritize the development and implementation of large-scale, multicenter RCTs to rigorously assess the efficacy and safety of probiotic supplementation in neonates. Ensuring standardization in probiotic composition, dosing strategies, and duration of administration is critical to enhance comparability across studies. Furthermore, long-term follow-up of neonatal growth trajectories, neurodevelopmental outcomes, and immune function is essential to fully determine the sustained benefits and potential risks of such interventions. Advancing our understanding of the neonatal gut microbiome and its interaction with probiotic interventions may also enable the identification of microbial profiles predictive of individual responsiveness. Such knowledge could facilitate the development of more personalized probiotic therapies. If promising strains identified in this meta-analysis are validated through high-quality, multicenter clinical trials, these findings may contribute substantially to the creation of evidence-based clinical guidelines for the use of probiotics in neonatal care.

## Figures and Tables

**Table 1:** Eligibility Criteria for Inclusion of Studies in the Meta-Analysis on Probiotic Supplementation and Neonatal Weight Gain.

Criteria	Inclusion	Exclusion
Population	Neonates (term and preterm) receiving probiotics, with a focus on weight gain outcomes.	Neonates with congenital or severe metabolic disorders affecting weight gain unrelated to probiotics.
Intervention	Probiotics administered (oral or enteral), including single-strain and multi-strain probiotics (e.g., *Lactobacillus reuteri*, *Bifidobacterium* species).	Studies not involving probiotics, or those without clear intervention details.
Outcome Measures	Neonatal weight gain measured at specific intervals (e.g., daily, weekly).	Studies that do not report weight gain as an outcome measure.
Study Type	Randomized controlled trials (RCTs) and observational studies.	Non-randomized studies without a clear outcome or methodology.
Age	Studies involving neonates and up to 6 months old.	Older than 6 months of age.
Publication Type	Published in peer-reviewed journals.	Articles not published in peer-reviewed journals.
Data Completeness	Studies with complete data on probiotic strain, dosage, and weight gain outcomes.	Studies with insufficient data on the probiotic strain, dosage, or weight gain outcomes.

**Table 2:** Summary of included studies reporting on neonatal weight gain and discharge weight (2011–2022).

No	Authors	Year	Region	Country	Intervention	Sample Size
1	Hays et al. [8]	2016	Lyon, Montpellier, and Bron	France	Probiotics (lactic + longum)	197
2	Totsu et al. [9]	2018	Tokyo	Japan	OLB6378 strain	207
3	Sari et al. [10]	2012	Ankara	Turkey	*Lactobacillus sporogenes*	174
4	Cui et al. [11]	2019	Shenyang	China	*Lactobacillus reuteri* DSM 17938	114
5	Oshiro et al. [12]	2019	Tokyo	Japan	*Bifidobacterium*	35
6	Totsu et al. [13]	2014	Tokyo	Japan	*Bifidobacterium bifidum* OLB6378	283
7	Shashidhar et al. [14]	2017	Bangalore	India	*Lactobacillus acidophilus*, *Lactobacillus rhamnosus*, *Bifidobacterium longum* and *Saccharomyces boulardii*	104
8	Xu et al. [15]	2016	Shenyang	China	*S. boulardii*	125
9	Moni et al. [16]	2017	Dhaka	Bangladesh	*Lactobacillus* and *Bifidobacterium*	65
10	Nouri et al. [17]	2015	Yazd	Iran	*Lactobacillus reuteri*	60
11	Patole et al. [18]	2014	Sydney	Australia	*B. breve* M16V	159
12	Wejryd et al. [19]	2019	Linköping	Sweden	*L. reuteri*	134
13	Van et al. [20]	2014	Tygerberg	South Africa	*Lactobacillus rhamnosus GG* and *Bifidobacterium*	110
14	Demirel et al. [21]	2013	Ankara	Turkey	*Saccharomyces boulardii*	271
15	Jacobs et al. [22]	2017	Melbourne	Australia	*B. infantis*, *S. thermophilus*, and *B. lactis*	1099
16	Serce et al. [23]	2013	Istanbul	Turkey	*Saccharomyces boulardii*	208
17	Al-Hosni et al. [24]	2012	Burlington	USA	*Lactobacillus rhamnosus GG* and *Bifidobacterium*	101
18	Sari et al. [25]	2011	Ankara	Turkey	*L. sporogenes*	221
19	Sowden et al. [26]	2022	Cape Town	South Africa	LabinicTM	200
20	Alshaikh et al. [27]	2022	Calgary	Canada	Multi-strain probiotics	62

## Data Availability

The original contributions presented in this study are included in the article. Further inquiries can be directed to the corresponding author.
